# Addendum to Proposal for Human Respiratory Syncytial Virus Nomenclature below the Species Level

**DOI:** 10.3201/eid2803.212438

**Published:** 2022-03

**Authors:** Ian G. Barr, Thomas C. Williams, Vahid Salimi, Ursula J. Buchholz

**Affiliations:** WHO Collaborating Centre for Reference and Research on Influenza, VIDRL, Doherty Institute, Melbourne, Victoria, Australia (I.G. Barr);; University of Edinburgh, Edinburgh, Scotland, UK (T.C. Williams);; Tehran University of Medical Sciences, Tehran, Iran (V. Salimi);; National Institute of Allergy and Infectious Diseases, National Institutes of Health, Maryland, USA (U. Buchholz)

**Keywords:** respiratory syncytial virus, HRSV, Human orthopneumovirus, human respiratory syncytial virus, isolates, nomenclature, respiratory infections, dual infections, specimens, strains, subspecies nomenclature, viruses

**To the Editor:** We previously proposed a nomenclature for human respiratory syncytial virus (HRSV) to standardize the sharing of viral isolates and sequences (*1*). This nomenclature was adopted by the World Health Organization’s Global RSV surveillance program and incorporated into the GISAID EpiRS platform (https://www.gisaid.org). One situation not covered in our proposal was when subtypes HRSV A and HRSV B coexist in the same clinical sample. Although this situation appears relatively infrequently, usually in <1% of HRSV-positive respiratory samples (*2*,*3*), some sources describe higher levels of codetection (e.g., 3.4% in a study from Senegal [*4*]). Dual infections may also be more frequently identified when subtype-specific PCR is introduced, as they have been in phase 2 of the World Health Organization RSV program (*5*,*6*). We offer an approach to clarify nomenclature in such instances of codetection. 

We recommend that the designations in the style of HRSV/A-B/Iran/1234/2021 be used in laboratory databases. However, the most important output from these samples is likely to be the genetic sequences. We recommend separate database submissions of the consensus sequences from HRSV A and HRSV B be designated, for example, HRSV/A/Iran/1234/2021 and HRSV/B/Iran/1234/2021, each having the same metadata and noting that both sequences came from the same clinical sample. Clearly identifying dual HRSV A and B infections will enable closer monitoring and, therefore, better understanding of the true frequency of these co-occurrences, of importance because dual infections raise interesting questions about illness severity compared with infection with HRSV A or B alone, duration of protection from reinfection, and factors modulating the frequency of dual infections. 

We also note that dual infections may raise technical difficulties, such as assignment of sequence reads to the correct subgroup. However, algorithms such as IRMA (image registration meta-algorithm) (*7*) that appear effective for sequencing approaches (e.g., Illumina, https://www.illumina.com) and long-read approaches (e.g., Oxford Nanopore, https://nanoporetech.com) might also be employed to ensure correct generation and assignment of HRSV A and B sequences from dual infections. Whereas co-infections with other respiratory pathogens are clearly recognized and well-studied, dual infections with HRSV A and B remain less so, but we are now well-positioned to identify these infections.
